# On Molecular Descriptors of Carbon Nanocones

**DOI:** 10.3390/biom8030092

**Published:** 2018-09-07

**Authors:** Waqas Nazeer, Adeel Farooq, Muhammad Younas, Mobeen Munir, Shin Min Kang

**Affiliations:** 1 Division of Science and Technology, University of Education, Lahore 54000, Pakistan; mmunir@ue.edu.pk; 2 Lahore Campus, COMSATS University Islamabad, Lahore 54000, Pakistan; adeelfarooq@cuilahore.edu.pk (A.F.); muhammadyounas@cuilahore.edu.pk (M.Y.); 3 Department of Mathematics and RINS, Gyeongsang National University, Jinju 52828, Korea; 4 Center for General Education, China Medical University, Taichung 40402, Taiwan

**Keywords:** M-polynomial, degree-based topological index, carbon nanocone

## Abstract

Many degree-based topological indices can be obtained from the closed-off M-polynomial of a carbon nanocone. These topological indices are numerical parameters that are associated with a structure and, in combination, determine the properties of the carbon nanocone. In this paper, we compute the closed form of the M-polynomial of generalized carbon nanocone and recover many important degree-based topological indices. We use software Maple 2015 (Maplesoft, Waterloo, ON, Canada) to plot the surfaces and graphs associated with these nanocones, and relate the topological indices to the structure of these nanocones.

## 1. Introduction

Chemical graph theory is a subject that connects mathematics, chemistry, and graph theory, and solves problems arising in chemistry mathematically. A topological index is a numeric number associated with a molecular graph that correlates certain physicochemical properties of chemical compounds. The topological indices are useful in the prediction of physicochemical properties and the bioactivity of the chemical compounds [1,2,3,4]. These indices capture the overall structure of the compound and predict chemical properties such as strain energy, heat formation, boiling points, etc.

Long computation is required to compute topological indices, and in order to simplify the computation of the degree indices, which form a subclass of degree-based topological indices of utmost importance, the M-polynomial was introduced in [5] by Deutsch and Klavžar. From the M-polynomial, one can recover nine degree-based topological indices. For this reason, the M-polynomial has been studied extensively in recent years, for example, Munir et al. computed M-polynomials for polyhex nanotubes, and recovered many important degree-based topological indices [6]. The M-polynomials for nanostar dendrimers were studied by Munir et al. in [7]. Munir et al. also studied the M-polynomials of titania nanotubes and its degree-based topological indices [8]. Ali et al. [9] studied zigzag and rhombic benzenoid systems. For more studies in this direction, see Munir et al. [10], Kwun et al. [11], Kang et al. [12], Ahmad et al. [13] and Kang et al. [14].

**Figure 1:**
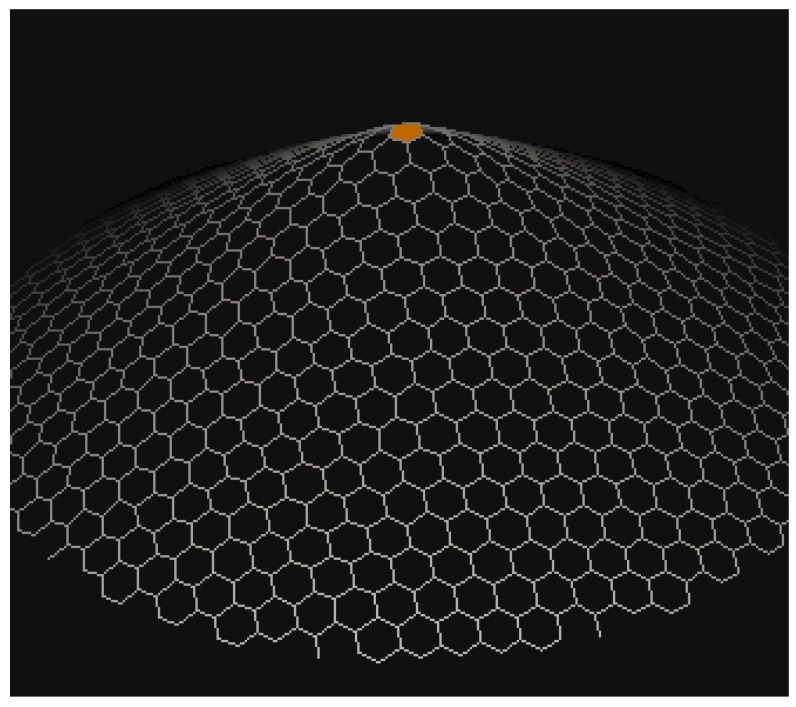
Carbon nanocone $CNC_{k}\left[n\right]$ for *k* = 5.

**Figure 2:**
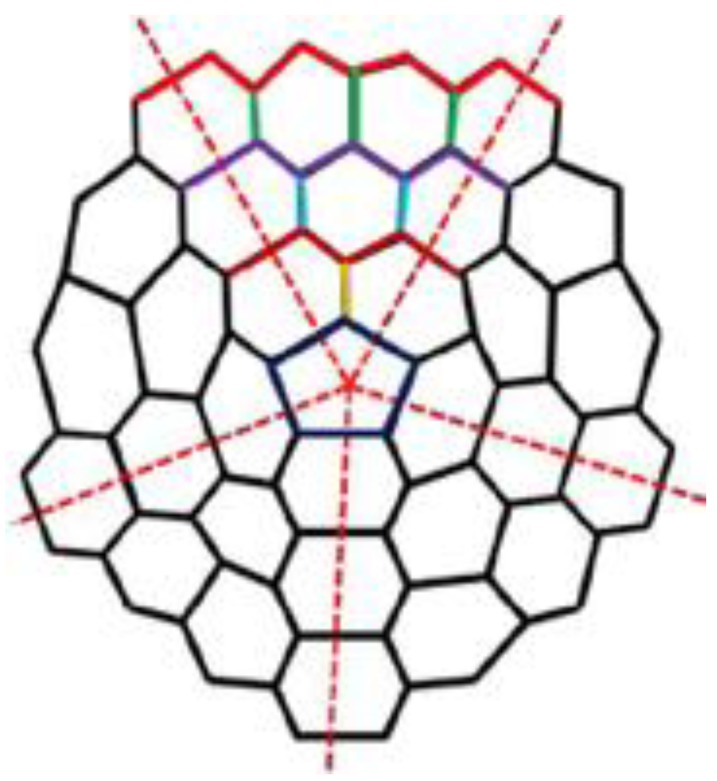
The molecular graph of $CNC_{k}\left[n\right]$ for *k* = 5.

Carbon nanocones have been observed since 1968 or even earlier [15] on the surface of naturally occurring graphite. The molecular graph of nanocones have conical structures with a cycle of length k at its core and n layers of hexagons placed at the conical surface around its center. The importance of carbon nanostructures is due to their potential use in many applications, including gas sensors, energy storage, nanoelectronic devices, biosensors, and chemical probes [16]. Carbon allotropes such as carbon nanocones and carbon nanotubes have been proposed as possible molecular gas storage devices [17]. More recently, carbon nanocones have gained increased scientific interest due to their unique properties and promising uses in many novel applications such as energy and hydrogen storage. Figure 1 and Figure 2 show carbon nanocones.

**Figure 3:**
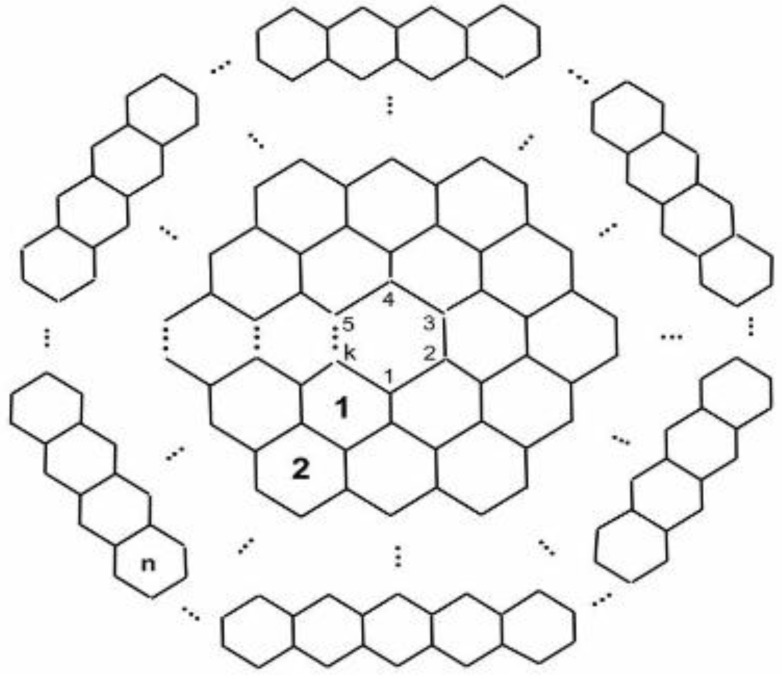
Carbon nanocone $CNC_{k}\left[n\right]$.

The molecular graph of $CNC_{k}\left[n\right]$ nanocones have conical structures with a cycle of length *k* at its core and *n* layers of hexagons placed at the conical surface around its center, as shown in the following Figure 3.

In the present report, we give a closed form of the M-polynomial of carbon nanocones. From the M-polynomial, we recover nine degree-based topological indices. In [18], Xu et al. computed the Hosoya polynomial and related distance-based indices for $CNC_{7}\left[n\right]$. In [19], Ghorbani et al. computed the Vertex PI, Szeged, and Omega polynomials of carbon nanocone $CNC_{4}\left[n\right]$. Similarly, many partial results regarding topological indices have been obtained for some particular classes of nanocones. However, we present some general results about complete families of nanocones. Our results present organized generalizations of many existing partial results.

.

## 2. Basic Definitions and Notions

Algebraic polynomials have many useful applications in chemistry. For instance, the Hosoya polynomial (also called the Wiener polynomial) [20] plays a vital role in determining distance-based topological indices. The M-polynomial [5], which was introduced in 2015, plays the same role in determining many degree-based topological indices [6,7,8,9,10,11,12,13,14].

Throughout this paper, *G* denotes connected graph, *V*(*G*) and *E*(*G*) denote the vertex set and the edge set, respectively, and *d_v_* denotes the degree of a vertex.

**Definition** **1.** *The M-polynomial of G is defined as:*

$$M\;\left(G,x,y\right)=\sum_{\delta \leq i\leq j\leq \Delta }m_{ij}\left(G\right)x^{i}y^{j},$$

*where $\delta =\mathrm{Min}\{d_{v}|v\in V\;(G)\},$$\Delta =\mathrm{Max}\{d_{v}|v\in V\;(G)\},$ and $m_{ij}(G)$ is the edge vu∈E(G) such that $\left\{d_{v},d_{u}\right\}=\left\{i,j\right\}$.*

The first well-known topological index was introduced by Wiener [21] when he was studying the boiling point of paraffin. He named it the path number, which is now known as the Wiener index [22,23]. Later, Randic defined the first degree-based topological index in 1975 [24]. The Randic index is denoted by $R_{-1/2}(G)$, and is defined as:

$$R_{-1/2}(G)=\sum_{uv\in E(G)}\frac{1}{\sqrt{d_{u}d_{v}}}.$$

In 1998, working independently, Bollobas and Erdos [25] and Amic et al. [26] proposed the generalized Randic index, which has been studied extensively by both chemists and mathematicians [27]. Many mathematical properties of the Randic index have been discussed [28]. For a detailed survey we refer to the monograph of Li and Gutman [29].

The general Randic index is defined as:

$$R_{\alpha }(G)=\sum_{uv\in E(G)}{\left(d_{u}d_{v}\right)}^{\alpha }.$$

Obviously $R_{-1/2}(G)$ is the particular case of $R_{\alpha }(G)$ when $\alpha =-\frac{1}{2}$.

Gutman and Trinajstic introduced the first Zagreb index and second Zagreb index, which are defined as: $M_{1}(G)=\sum_{uv\in E(G)}\left(d_{u}+d_{v}\right)$ and $M_{2}(G)=\sum_{uv\in E(G)}\left(d_{u}\times d_{v}\right)$, respectively. The second modified Zagreb index was defined as:

$${}^{m}M_{2}\left(G\right)=\sum_{uv\in E(G)}\frac{1}{d(u)d(v)}.$$

For detail about these indices, we refer Nikolić et al. [30], Gutman and Das [31], Das and Gutman [32] and Trinajstić et al. [33] to the readers.

The symmetric division index is defined as:

$$SDD\left(G\right)=\sum_{uv\in E(G)}\left\{\frac{\min(d_{u},d_{v})}{\max(d_{u},d_{v})}+\frac{\max(d_{u},d_{v})}{\min(d_{u},d_{v})}\right\}.$$

Other well-known topological indices are the harmonic index, $H(G)=\sum_{vu\in E(G)}\frac{2}{d_{u}+d_{v}}.$, the inverse sum index, $I(G)=\sum_{vu\in E(G)}\frac{d_{u}d_{v}}{d_{u}+d_{v}}$, and the augmented Zagreb index [34,35]:

$$A(G)=\sum_{vu\in E(G)}{\left\{\frac{d_{u}d_{v}}{d_{u}+d_{v}-2}\right\}}^{3}.$$

The following Table 1 relates some well-known degree-based topological indices with the M-polynomial [5].

where:

$$\begin{array}{l} D_{x}=x\frac{\partial (f\left(x,y\right)}{\partial x},\;D_{y}=y\frac{\partial (f\left(x,y\right)}{\partial y},\;S_{x}=\overset{x}{\underset{0}{\int }}\frac{f\left(t,y\right)}{t}dt,S_{y}=\overset{y}{\underset{0}{\int }}\frac{f\left(x,t\right)}{t}dt,\;J\left(f\left(x,y\right)\right)=f\left(x,x\right),\\ Q_{\alpha }\left(f\left(x,y\right)\right)=x^{\alpha }f\left(x,y\right). \end{array}$$

## 3. Results

In this section, we give our computational results.

**Theorem** **1.** *Let $CNC_{k}\left[n\right]$ be the graph of carbon nanocones. Then, the M-polynomial of $CNC_{k}\left[n\right]$ is:*

$$M\left(CNC_{k}\left[n\right],x,y\right)=kx^{2}y^{2}+2knx^{2}y^{3}+\frac{kn}{3}\left(3n+1\right)x^{3}y^{3}.$$

**Proof.**  Let $CNC_{k}\left[n\right]$ be the graph of nanocones. From the graph of $CNC_{k}\left[n\right]$ nanocones, we can see that there are two partitions, $V_{\left\{3\right\}}=\{v\in V\left(CNC_{k}\left[n\right]\right)|d_{v}=3\}$ and $V_{\left\{2\right\}}=\{v\in V\left(CNC_{k}\left[n\right]\right)|d_{v}=2\}.$ The edge set of the $CNC_{k}\left[n\right]$ can be partitions as follows:

$$E_{\left\{2,2\right\}}=\{e=uv\in E\left(CNC_{k}\left[n\right]\right)|d_{u}=2\&d_{v}=2\},$$

$$E_{\left\{2,3\right\}}=\{e=uv\in E\left(CNC_{k}\left[n\right]\right)|d_{u}=2\&d_{v}=3\}$$

and:

$$E_{\left\{3,3\right\}}=\{e=uv\in E\left(CNC_{k}\left[n\right]\right)|d_{u}=d_{v}=3\}$$

From the molecular graph of $CNC_{k}\left[n\right]$, we can observe that $\left|E_{\left\{2,2\right\}}\right|=k,$ $\left|E_{\left\{2,3\right\}}\right|=2kn$, and $\left|E_{\left\{3,3\right\}}\right|=\frac{kn}{3}\left(3n+1\right).$

**Figure 4:**
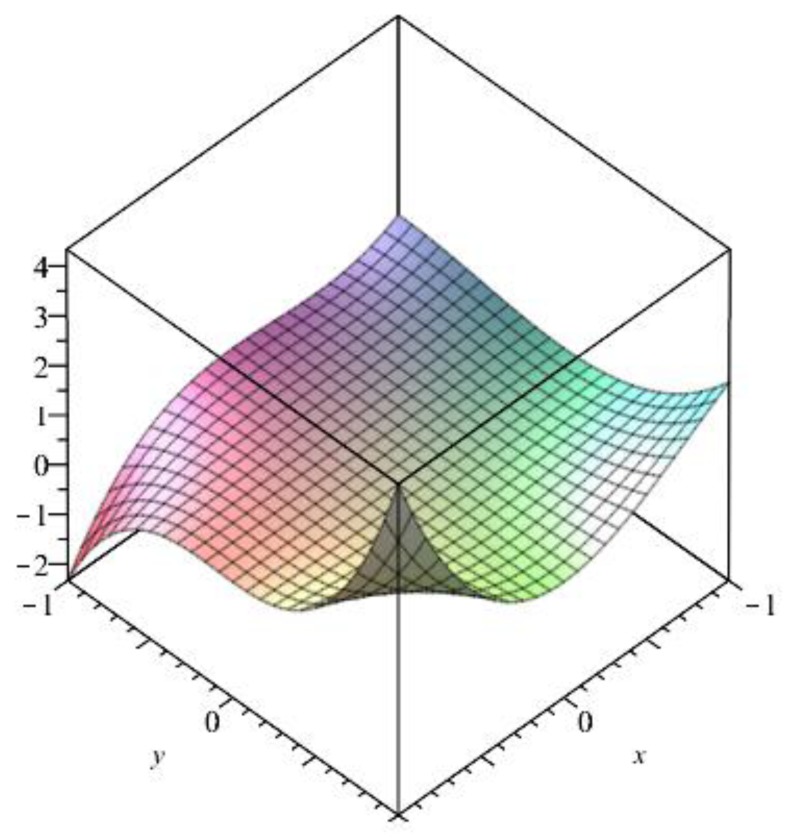
The 3D plot for the M-polynomial of $CNC_{k}\left[n\right]$ for *k* = *n* = 1.

Thus, by Definition 1, the M-polynomial of $CNC_{k}\left[n\right]$ (Figure 4) is:

$$\begin{array}{ll} M\left(CNC_{k}\left[n\right];x,y\right) & =\sum_{i\leq j}m_{ij}\left(CNC_{k}\left[n\right]\right)x^{i}y^{j},\\ & =\sum_{2\leq 2}m_{22}\left(CNC_{k}\left[n\right]\right)x^{2}y^{2}+\sum_{2\leq 3}m_{23}\left(CNC_{k}\left[n\right]\right)x^{2}y^{3}+\sum_{3\leq 3}m_{33}\left(CNC_{k}\left[n\right]\right)x^{3}y^{3},\\ & =\sum_{uv\in E_{\left\{2,2\right\}}}m_{22}\left(CNC_{k}\left[n\right]\right)x^{2}y^{2}+\sum_{uv\in E_{\left\{2,3\right\}}}m_{23}\left(CNC_{k}\left[n\right]\right)x^{2}y^{3}+\sum_{uv\in E_{\left\{3,3\right\}}}m_{33}\left(CNC_{k}\left[n\right]\right)x^{3}y^{3},\\ & =\left|E_{\left\{2,2\right\}}\right|x^{2}y^{2}+\left|E_{\left\{2,3\right\}}\right|x^{2}y^{3}+\left|E_{\left\{3,3\right\}}\right|x^{3}y^{3},\\ & =kx^{2}y^{2}+2knx^{2}y^{3}+\frac{kn}{3}\left(3n+1\right)x^{3}y^{3}. \end{array}$$

 ☐

**Theorem** **2.** *Let $CNC_{k}\left[n\right]$ be the graph of carbon nanocones. Then:*

1. $M_{1}\left(CNC_{k}\left[n\right]\right)=6kn^{2}+12kn+4k.$
2. $M_{2}\left(CNC_{k}\left[n\right]\right)=9kn^{2}+15kn+4k.$
3. ${}^{m}M_{2}\left(CNC_{k}\left[n\right]\right)=\frac{1}{9}kn^{2}+\frac{10}{27}kn+\frac{1}{4}k.$
4. $R_{\alpha }\left(CNC_{k}\left[n\right]\right)=k2^{2\alpha }+kn\left(n3^{2\alpha }+2^{2\alpha +1}+3^{2\alpha -1}\right).$
5. $R_{\alpha }\left(CNC_{k}\left[n\right]\right)=k4^{-\alpha }+2kn6^{-\alpha }+\frac{1}{3}kn(3n+1)9^{-\alpha }.$
6. $SDD\left(CNC_{k}\left[n\right]\right)=2kn^{2}+5kn+2k.$
7. $H\left(CNC_{k}\left[n\right]\right)=\frac{1}{3}kn^{2}+\frac{41}{45}kn+\frac{1}{2}k.$
8. $I\left(CNC_{k}\left[n\right]\right)=\frac{3}{2}kn^{2}+\frac{29}{10}kn+k.$
9. $A\left(CNC_{k}\left[n\right]\right)=\frac{729}{64}kn^{2}+\frac{1267}{64}kn+8k.$

**Proof.**  Let $M\left(CNC_{k}\left[n\right];x,y\right)=f\left(x,y\right)=kx^{2}y^{2}+2knx^{2}y^{3}+\frac{kn}{3}\left(3n+1\right)x^{3}y^{3},$

$$D_{x}\left(f\left(x,y\right)\right)=2kx^{2}y^{2}+4knx^{2}y^{3}+kn\left(3n+1\right)x^{3}y^{3},\;$$

$$D_{y}\left(f\left(x,y\right)\right)=2kx^{2}y^{2}+6knx^{2}y^{3}+kn\left(3n+1\right)x^{3}y^{3},\;$$

$$S_{x}\left(f\left(x,y\right)\right)=\frac{k}{2}x^{2}y^{2}+knx^{2}y^{3}+\frac{kn}{9}\left(3n+1\right)x^{3}y^{3},\;$$

$$S_{y}\left(f\left(x,y\right)\right)=\frac{k}{2}x^{2}y^{2}+\frac{2}{3}knx^{2}y^{3}+\frac{kn}{9}\left(3n+1\right)mx^{3}y^{3},\;$$

$$D_{x}^{\alpha }D_{y}^{\alpha }(f(x,y))=2^{2\alpha }kx^{2}y^{2}+2^{\alpha +1}3^{\alpha }knx^{2}y^{3}+3^{2\alpha -1}kn\left(3n+1\right)x^{3}y^{3},\;$$

$$S_{x}^{\alpha }S_{y}^{\alpha }(f(x,y))=\frac{k}{2^{2\alpha }}x^{2}y^{2}+\frac{1}{2^{\alpha -1}3^{\alpha }}knx^{2}y^{3}+\frac{kn}{3^{2\alpha +1}}\left(3n+1\right)x^{3}y^{3}\;$$

$$J\left(f\left(x,y\right)\right)=kx^{4}+2knx^{5}+\frac{kn}{3}\left(3n+1\right)x^{6},\;$$

$$S_{x}J\left(f\left(x,y\right)\right)=\frac{k}{4}x^{4}+\frac{2}{5}knx^{5}+\frac{kn}{18}\left(3n+1\right)x^{6},\;$$

$$D_{x}D_{y}\left(f\left(x,y\right)\right)=4kx^{2}y^{2}+12knx^{2}y^{3}+3kn\left(3n+1\right)x^{3}y^{3},\;$$

$$JD_{x}D_{y}\left(f\left(x,y\right)\right)=4kx^{4}+12knx^{5}+3kn\left(3n+1\right)x^{6},\;$$

$$S_{x}JD_{x}D_{y}\left(f\left(x,y\right)\right)=kx^{4}+\frac{12}{5}knx^{5}+\frac{1}{2}kn\left(3n+1\right)x^{6},\;$$

$$D_{y}^{3}\left(f\left(x,y\right)\right)=8kx^{2}y^{2}+54knx^{2}y^{3}+9kn\left(3n+1\right)x^{3}y^{3},\;$$

$$D_{x}^{3}D_{y}^{3}\left(f\left(x,y\right)\right)=64kx^{2}y^{2}+432knx^{2}y^{3}+243kn\left(3n+1\right)x^{3}y^{3},\;$$

$$JD_{x}^{3}D_{y}^{3}\left(f\left(x,y\right)\right)=64kx^{4}+432knx^{5}+243kn\left(3n+1\right)x^{6},\;$$

$$Q_{-2}JD_{x}^{3}D_{y}^{3}\left(f\left(x,y\right)\right)=64kx^{2}+432knx^{3}+243kn\left(3n+1\right)x^{4},\;$$

$$S_{x}^{3}Q_{-2}JD_{x}^{3}D_{y}^{3}\left(f\left(x,y\right)\right)=8kx^{2}+16knx^{3}+\frac{243}{64}kn\left(3n+1\right)x^{4}.$$

 ☐

**Figure 5:**
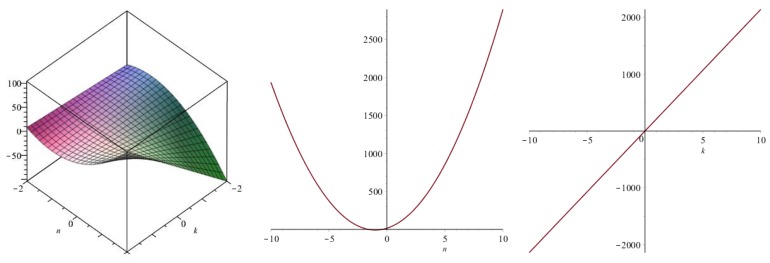
Plots for the first Zagreb index (left for arbitrary *n* and *k*, middle for *k* = 4, and right for *n* = 5).

**Figure 6:**
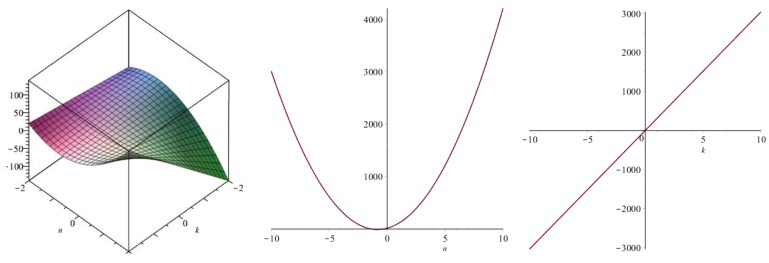
Plots for the second Zagreb index (left for arbitrary *n* and *k*, middle for *k* = 4, and right for *n* = 5).

**Figure 7:**
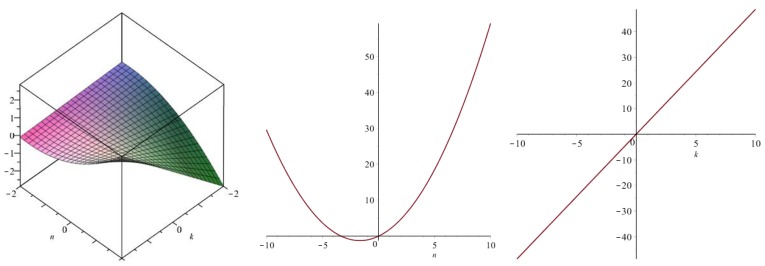
Plots for the modified second Zagreb index (left for arbitrary *n* and *k*, middle for *k* = 4, and right for *n* = 5).

**Figure 8:**
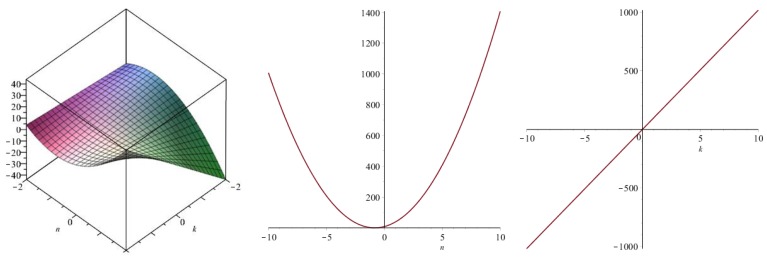
Plots for the Randic index (left for arbitrary *n* and *k*, middle for *k* = 4, and right for *n* = 5).

**Figure 9:**
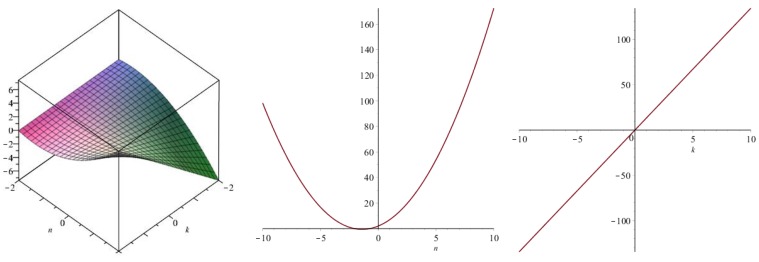
Plots for the inverse Randic index (left for arbitrary *n* and *k*, middle for *k* = 4, and right for *n* = 5).

**Figure 10:**
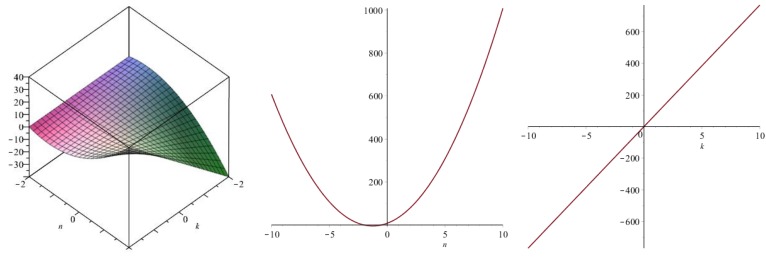
Plots for the symmetric division index (left for arbitrary *n* and *k*, middle for *k* = 4, and right for *n* = 5).

**Figure 11:**
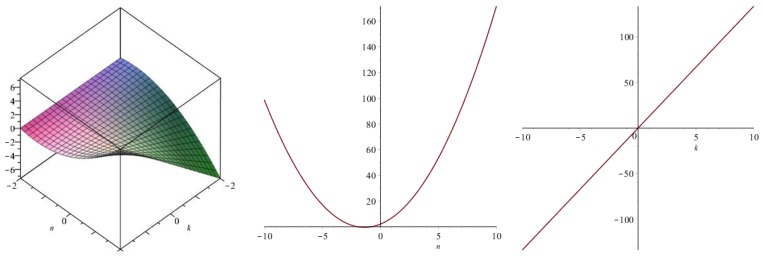
Plots for the harmonic index (left for arbitrary *n* and *k*, middle for *k* = 4, and right for *n* = 5).

**Figure 12:**
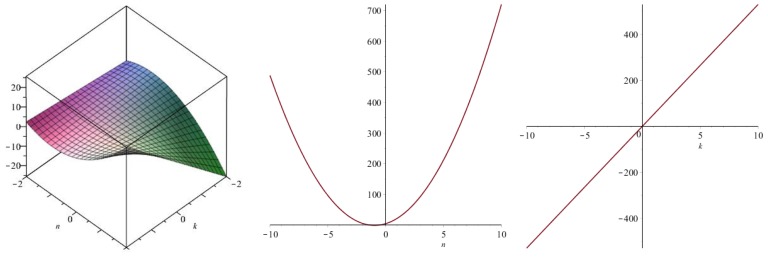
Plots for the inverse sum index (left for arbitrary *n* and *k*, middle for *k* = 4, and right for *n* = 5).

**Figure 13:**
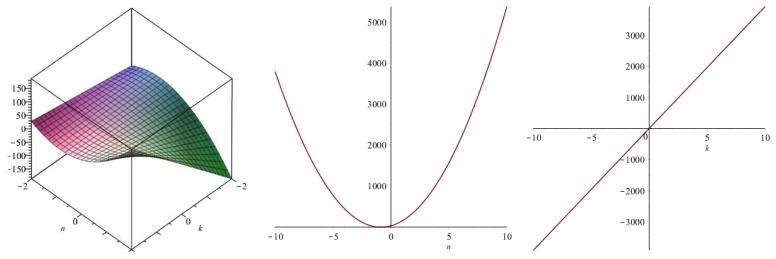
Plots for the augmented Zagreb index (left for arbitrary *n* and *k*, middle for *k* = 4, and right for *n* = 5).

Now we recover degree-based topological indices by using Table 1. The Figure 5, Figure 6, Figure 7, Figure 8, Figure 9, Figure 10, Figure 11, Figure 12 and Figure 13 show the relations of different topological indices with values of *k* and *n*. It is noticeable that all of the above discussed topological indices vary quadratically with *n*, and linearly with *k*.

1. $M_{1}\left(CNC_{k}\left[n\right]\right)=\left(D_{x}+D_{y}\right)\left(f\left(x,y\right)\right)\left(M\left(CNC_{k}\left[n\right]\right)x,y\right){|}_{x=y=1}=\frac{k}{2}\left(18n^{2}+26n+10\right).$

2. $M_{2}\left(CNC_{k}\left[n\right]\right)=\left(D_{x}D_{y}\right)\left(f\left(x,y\right)\right)\left(M\left(CNC_{k}\left[n\right]\right)x,y\right){|}_{x=y=1}=\frac{k^{2}}{4}\left(81n^{4}+234n^{3}+255n^{2}+126n+24\right).$

3. ${}^{m}M_{2}\left(CNC_{k}\left[n\right]\right)=\left(S_{x}S_{y}\right)\left(f\left(x,y\right)\right){|}_{x=y=1}=\frac{1}{9}kn^{2}+\frac{10}{27}kn+\frac{1}{4}k.$

4. $R_{\alpha }\left(CNC_{k}\left[n\right]\right)=D_{x}^{\alpha }D_{y}^{\alpha }f\left(x,y\right){|}_{x=y=1}=k2^{2\alpha }+kn\left(n3^{2\alpha }+2^{2\alpha +1}+3^{2\alpha -1}\right).$

5. $R_{\alpha }\left(CNC_{k}\left[n\right]\right)=\left(S_{x}^{\alpha }S_{y}^{\alpha }\right)\left(f\left(x,y\right)\right){|}_{x=y=1}=k4^{-\alpha }+2kn6^{-\alpha }+\frac{1}{3}kn(3n+1)9^{-\alpha }.$

6. $SDD\left(CNC_{k}\left[n\right]\right)=\left(D_{x}S_{y}+D_{y}S_{x}\right)\left(f\left(x,y\right)\right){|}_{x=y=1}=2kn^{2}+5kn+2k.$

7. $H\left(CNC_{k}\left[n\right]\right)=2S_{x}J\left(f\left(x,y\right)\right){|}_{x=1}=\frac{1}{3}kn^{2}+\frac{41}{45}kn+\frac{1}{2}k.$

8. $I\left(CNC_{k}\left[n\right]\right)=S_{x}JD_{x}D_{y}\left(f\left(x,y\right)\right){|}_{x=1}=\frac{3}{2}kn^{2}+\frac{29}{10}kn+k.$

9. $A\left(CNC_{k}\left[n\right]\right)=S_{x}^{3}Q_{-2}JD_{x}^{3}D_{y}^{3}\left(f\left(x,y\right)\right){|}_{x=1}=\frac{729}{64}kn^{2}+\frac{1267}{64}kn+8k.$

## 4. Conclusions

The closed form of the M-polynomials of all of the carbon nanocones is computed. This polynomial generates a lot of information about degree-based topological descriptors, which are actually graph invariants. These indices, in combination, determine the properties of nanocones. The topological indices calculated in this paper are important for guessing the physicochemical properties of understudy chemical compounds. For example, the Randić index is a topological descriptor that has been connected with numerous substance qualities of atoms, and has been found to be parallel to processing the boiling point and Kovats constants of the particles. To associate with certain physicochemical properties, the GA index has a very preferable prescient control over the prescient intensity of the Randić index. The first and second Zagreb indexes were found to calculate the aggregate π-electron vitality of the atoms inside specific surmised articulations. These are among the graph invariants, which were proposed for the estimation of the skeleton of the spreading of the carbon molecule. To calculate the distance-based topological indices of understudied nanocones is an interesting problem that is worthy of further investigation.

## Figures and Tables

**Table 1:** Derivation of some degree-based topological indices from the M-polynomial.

Topological Index	Derivation from M(G;x,y)
First Zagreb	$(D_{x}+D_{y})\left(M\left(G;x,y\right)\right){|}_{x=y=1}$
Second Zagreb	$(D_{x}D_{y})\left(M\left(G;x,y\right)\right){|}_{x=y=1}$
Second Modified Zagreb	$(S_{x}S_{y})\left(M\left(G;x,y\right)\right){|}_{x=y=1}$
General Randić Index	$(D_{x}^{\alpha }D_{y}^{\alpha })\left(M\left(G;x,y\right)\right){|}_{x=y=1}$
General inverse Randić Index	$(S_{x}^{\alpha }S_{y}^{\alpha })\left(M\left(G;x,y\right)\right){|}_{x=y=1}$
Symmetric Division Index	$(D_{x}S_{y}+S_{x}D_{y})\left(M\left(G;x,y\right)\right){|}_{x=y=1}$
Harmonic Index	$2\;S_{x}\;J\;{\left(M(G\;;\;x,y)\right)}_{x=1}$
Inverse sum Index	$S_{x}\;J\;D_{x}\;D_{y}{\left(M(G\;;\;x,y)\right)}_{x=1}$
Augmented Zagreb Index	$S_{x}{}^{3}\;Q_{-2}\;J\;D_{x}{}^{3}D_{y}{}^{3}{\left(M(G\;;\;x\;,\;y)\right)}_{x=1}$
